# Development of a High-Throughput Screening Assay to Identify Inhibitors of the SARS-CoV-2 Guanine-N7-Methyltransferase Using RapidFire Mass Spectrometry

**DOI:** 10.1177/24725552211000652

**Published:** 2021-03-16

**Authors:** Lesley-Anne Pearson, Charlotte J. Green, De Lin, Alain-Pierre Petit, David W. Gray, Victoria H. Cowling, Euan A. F. Fordyce

**Affiliations:** 1 Drug Discovery Unit, School of Life Sciences, University of Dundee, Dundee, UK; 2 Centre for Gene Regulation and Expression, School of Life Sciences, University of Dundee, Dundee, UK

**Keywords:** nsp14, COVID-19, SARS-CoV-2, antiviral

## Abstract

Severe acute respiratory syndrome coronavirus-2 (SARS-CoV-2) represents a significant threat to human health. Despite its similarity to related coronaviruses, there are currently no specific treatments for COVID-19 infection, and therefore there is an urgent need to develop therapies for this and future coronavirus outbreaks. Formation of the cap at the 5′ end of viral RNA has been shown to help coronaviruses evade host defenses. Nonstructural protein 14 (nsp14) is responsible for N7-methylation of the cap guanosine in coronaviruses. This enzyme is highly conserved among coronaviruses and is a bifunctional protein with both N7-methyltransferase and 3′-5′ exonuclease activities that distinguish nsp14 from its human equivalent. Mutational analysis of SARS-CoV nsp14 highlighted its role in viral replication and translation efficiency of the viral genome. In this paper, we describe the characterization and development of a high-throughput assay for nsp14 utilizing RapidFire technology. The assay has been used to screen a library of 1771 Food and Drug Administration (FDA)-approved drugs. From this, we have validated nitazoxanide as a selective inhibitor of the methyltransferase activity of nsp14. Although modestly active, this compound could serve as a starting point for further optimization.

## Introduction

Since emerging in Wuhan, China, in December 2019, severe acute respiratory syndrome coronavirus-2 (SARS-CoV-2, has led to more than 95 million confirmed cases of COVID-19 and more than 2 million deaths worldwide (numbers accurate as of January 19, 2021).^1^ COVID-19 is a contagious disease that generally causes mild symptoms that include fever, a dry cough, and tiredness. Approximately 10%–15% of cases progress to severe disease, and about 5% of those infected become critically ill.^2^ Currently, only supportive care is available. Multiple COVID-19 variants are circulating globally (including variants in the UK, South Africa, and Brazil) that appear to spread more easily and quickly than other variants. Currently, there is no evidence that these variants cause more severe illness or increased risk of death. However, this has increased the burden placed on healthcare systems around the world, and there is a pressing need for the discovery and development of therapeutic agents to treat this disease. The quickest way to achieve this is to repurpose approved drugs developed for other uses, and thereby take advantage of existing information on human pharmacology and toxicology to enable rapid clinical trials.

SARS-CoV-2 is a positive-sense single-stranded RNA virus belonging to the beta genus of the Coronaviridae family.^3^ Like all viruses, SARS-CoV-2 is fully reliant on the translation machinery of its host cells to translate RNA into the proteins that are essential for its survival. In order to protect itself from cellular innate immunity, viral mRNA of SARS-CoV-2 possesses a cap structure at the 5′-end of the polynucleotide, consisting of an *N*-methylated guanosine triphosphate and a C2′-*O*-methyl-ribosyladenine.^4^ Similar cap structures exist on eukaryotic cellular mRNAs;^5^ however, the cap is installed on RNA during transcription in the nucleus, and therefore coronaviruses, such as SARS-CoV-2, do not have access to the mammalian capping enzymes. Instead, coronaviruses encode their own cap-synthesizing enzymes.^6^ Four nonstructural proteins (nsp) are thought to be involved in the capping process: nsp10, nsp13, nsp14, and nsp16. The primary function of nsp13 is the unwinding of viral RNA during replication,^7^ but it also has 5′-RNA triphosphatase activity;^8^ thereby it is responsible for cleaving monophosphate at the 5′-end of the polynucleotide. The protein responsible for initial cap creation, the guanylyltransferase, is not known. Nsp14 and nsp16 are responsible for the methylation of the cap on the guanine of the GTP and the C2′-hydroxyl group of the following nucleotide, respectively.^9–13^ Both are *S*-adenosylmethionine (SAM)-dependent methyltransferases (MTases). Nsp10 is known to interact with nsp14 and nsp16 and regulate their respective ExoN and 2′-*O*-MTase activities.^12,14^ Nsp10 is an allosteric regulator that stabilizes nsp16.^11^ In addition, nsp16 binding extends and narrows the RNA-binding groove that accommodates the RNA substrate, thereby promoting the RNA- and SAM-binding capabilities of nsp16.

Nsp14 is well conserved within the Coronaviridae family.^15^ It is a bifunctional protein with both N7-MTase and 3′- to 5′-exonuclease (ExoN) activities. While the association of nsp10 with nsp14 stimulates the ExoN activity of nsp14 by >35-fold,^16^ there is no evidence that it has any effect on N7-MTase activity. The specific role of N7-MTase activity in virus replication is supported by reverse genetics. Chen et al. have introduced point mutations into a SARS-CoV-1 replicon carrying a luciferase reporter.^9^ A D331A mutation (MTase domain) led to reduction of luciferase activity by 90% and the copy number of subgenomic RNA by 81%. More recently, mutations in murine hepatitis virus nsp14 at G332 within the MTase domain resulted in delayed replication kinetics and decreased peak titers relative to wild-type.^17^ In addition, replication of nsp14 G332A virus was diminished following treatment of cells with IFN-β, and nsp14 G332A genomes were translated less efficiently both in vitro and during viral infection. Taken together, these results demonstrate that the N7-MTase activity of nsp14 plays an important role in viral replication.

Nsp14 N7-MTase is an attractive target for antiviral strategies. Although its overall structure is similar to the human homolog, RNA guanine-N7-methyltransferase (RNMT), there is very little sequence conservation in the active site (7% sequence identity between RNMT and SARS-CoV-2 nsp14 was calculated using SuperPose;^18^ RNMT (PDB:5E8J) and a SARS-CoV-2 homology model based on the SARS-CoV-1 nsp14 structure (PDB:5C8U) were used for this comparison), suggesting that molecules with selectivity for the viral protein should be achievable. The ligandability of the enzyme with small molecules has been demonstrated with known MTase inhibitors. In vitro assays revealed that *S*-adenosyl-l-homocysteine (the by-product of the methylation reaction), sinefungin, and aurintricarboxylic acid (ATA) efficiently inhibit nsp14 N7-MTase activity with IC_50_ values of 16 µM, 496 nM, and 6.4 µM, respectively.^19^ ATA was also shown to limit SAR-CoV replication in infected cells.^20^ Taken together, nsp14 represents a novel, viable drug target for the treatment of COVID-19 and potentially more widely across the Coronaviridae family. Herein, we report the development of a mass spectrometry (MS)-based high-throughput screen in order to identify small-molecule inhibitors of the N7-MTase activity of SARS-CoV-2 nsp14. Using this technology, we screened a library composed of 1771 Food and Drug Administration (FDA)-approved small molecules.

## Materials and Methods

All aqueous solutions were prepared with deionized water (Millipore, Watford, Hertfordshire, UK). All reagents were purchased from Sigma Aldrich (Gillingham, Dorset, UK) unless otherwise stated. Full-length SARS-CoV-2 nsp14 protein (DU66418) was supplied by the MRC-PPU (Dundee, UK). Nsp14 was cloned in fusion with a cleavable N-terminal GST fusion in a pGEX6P1 vector and expressed in *Escherichia coli*. Nsp14 was purified by batch purification using GSH-Sepharose beads and the tag was cleaved by PreScission protease. Cleaved nsp14 was delivered in 50 mM Tris, pH 7.5, 150 mM NaCl, 270 mM sucrose, 0.1 mM EGTA, 0.03% Brij-35, 0.1% β-mercaptoethanol at 1.23 mg/mL. Full-length human RNMT:RAM (1–90) was coexpressed using conditions developed internally.

### Description of the Screening Library

The DiscoveryProbe FDA-approved drug library (https://www.apexbt.com) is a unique collection of 1771 small-molecule, FDA-approved drugs with known bioavailability and safety data in humans.

### Nsp14 RapidFire MS High-Throughput Screening Assay

An endpoint 384-well plate assay was developed to assess nsp14 activity. Briefly, enzyme and substrates, SAM (*S*-(5′-adenosyl)-l-methionine chloride hydrochloride [Cayman Chemical, Ann Arbor, MI] and cap G(5′)ppp(5′)G sodium salt [New England Biolabs, Ipswich, MA]) were incubated to allow the reaction to take place, and then the product was quantified using MS. Assays were performed in 384-well, clear, flat-bottom plates (Greiner 781101) to a final volume of 20 µL. Components were diluted in buffer (20 mM Tris, pH 8.0, 50 mM NaCl) containing 1 mM TCEP (Thermo Scientific, Waltham, MA), 0.1 mg/mL bovine serum albumin, 0.005% Nonidet P40 (Roche), and 3 mM MgCl_2_. In the assay, 5 nM nsp14 was incubated with 1 µM SAM and 0.7 µM cap (FAC). Following a 60 min incubation, the reaction was quenched using 1% formic acid (VWR, Radnor, PA) containing 0.03 µg/mL *S*-adenosylhomocysteine-d4 (d4SAH; Cambridge Bioscience, Cambridge, UK) and loaded on the RapidFire system by aspiration for 600 ms using the Agilent RapidFire 365 high-throughput system with integrated solid-phase extraction (SPE) interfaced with the Agilent 6740 triple quadrupole mass spectrometer. The sample was then automatically loaded onto a C18 Type C SPE cartridge (Agilent Technologies), and buffer salts and protein matrix were removed from the sample by washing the cartridge with the load solution (water containing 0.1% trifluoroacetic acid [TFA]) at a flow rate of 1.5 mL/min for 5000 ms. The retained and purified analytes were eluted from the cartridge with the elution solution (acetonitrile: water [9:1, v/v] containing 0.1% TFA) at 1.25 mL/min for 5000 ms and directed to the mass spectrometer. The cartridge was reequilibrated with load solution at 1.5 mL/min for 500 ms.

Both *S*-adenosylhomocysteine (SAH) and d4SAH were assessed using multiple selected reaction monitoring (MRM) transitions of 385.1/134 for SAH and 389.2/135.9 for d4SAH. The dwell time was 50 ms for each transition. The fragmentor voltage was set to 120 V for SAH and 100 for d4SAH, the collision energy to 12 V for SAH and 14 V for d4SAH, the cell accelerator voltage to 5 V, and the delta electron multiplier voltage to 200 V. The mass spectrometer was operated with a gas temperature of 350 °C, gas flow rate of 7 L/min, nebulizer pressure of 40 psi, and capillary voltage of 3000 V. The areas under the daughter ion peaks of SAH and d4SAH were integrated using RapidFire QQQ Quantitative Analysis software (Agilent Technologies), and the area ratios of the SAH to the internal standard d4SAH were used for quantitation. A representative chromatograph showing a time course of nsp14 in this assay has been included in the supplemental files (**Suppl. Fig. S1**).

### SAM Enzyme Kinetics

To determine the kinetics for SAM, a twofold serial dilution was performed from a top concentration of 20 µM, with 20 µM cap. Reaction was initiated by addition of 10 nM nsp14 at multiple time points, and the plate was read using RapidFire. Data collected from the experiment were analyzed to calculate initial velocities and obtain *K*_M_ values using the Michaelis–Menten equation (SigmaPlot version 14.0; Systat Software, Inc., San Jose, CA). *K*_M_^app^ values are presented as the mean of three independent experiments with the 95% CI.

### Cap Enzyme Kinetics

To determine the kinetics for cap, a twofold serial dilution was performed from a top concentration of 20 µM, with 20 µM SAM. Reaction was initiated by addition of 10 nM nsp14 at multiple time points, and the plate was read using RapidFire. Data were analyzed as described previously.

### Assay Development

An optimal nsp14 concentration was established using a range of concentrations starting from 15 nM, with incubation times up to 120 min to establish the most appropriate incubation time when using established screening concentrations of substrates. Assay DMSO (VWR) tolerance was tested from 5%, added using the Preddator Reagent Dispenser (Redd and Whyte, London, UK) with a single incubation time of 60 min. The impact of addition of nsp10 on apparent nsp14 activity was assessed by titrating nsp10 into the assay from a top concentration of 20 nM, using previously established screening concentrations of substrates and nsp14, with incubation times up to 60 min. Reagent stability experiments were carried out whereby reactions were started 0, 1, 2, and 4 h after reagent preparation, with and without the addition of nsp10 at equimolar concentrations to nsp14. All reagents were maintained at room temperature throughout the course of the experiment, and the assay was run for a single incubation time of 60 min. Assay robustness was assessed by performing a mock screen, consisting of two empty 384-well plates, one of which contained compounds from our assay interference set. Assay performance was further assessed by screening the SAM mimetic, sinefungin, at a 10-point, 1:2 serial dilution from 100 µM. For calibration curves, SAH was spiked into the assay at concentrations from 0.017 to 10 µM in assay buffer and quenching solution containing 0.08 µM internal standard (d4 SAH) and measured by RapidFire MS/MS (**Suppl. Fig. S2**).

### Compound Testing

For compound testing, DMSO stocks of the FDA library compounds and DMSO backfill were dispensed (0.02 μL of constant total volume, 0.1% of the final assay volume for single-point testing, 0.2 µL of constant total volume, 1% of the final assay volume for 10-point dose–response testing) into dry 384-well assay plates (Greiner 781101) using the Echo 550 acoustic dispenser (Labcyte, Sunnyvale, CA), giving a final assay concentration of 10 µM. The enzyme solution (10 μL) was added to predispensed compounds first, and then addition of the substrate solution (10 μL) initiated the reaction. The inhibitory activity was calculated using the peak area ratio, which is the reaction product (SAH) divided by its internal standard (d4SAH). We defined the peak area ratio of the reaction without enzyme as 100% inhibitory activity and that of the complete reaction mixture as 0% inhibitory activity.

Curve fitting and calculations of IC_50_ values were undertaken using ActivityBase XE version 9.2.0.106 from IDBS. A four-parameter logistics dose–response curve (model 203) was utilized with prefit for all four parameters. Unless otherwise stated, all IC_50_ values are presented with 95% confidence intervals with the associated *n* values.

### In Vitro Cap Guanosine N7-Methyltransferase Assay

Cap MTase assays were performed according to Cowling, with minor alterations.^21^ In brief, 33 nM recombinant SARS-CoV-2 nsp14 was incubated with a stated concentration of nitazoxanide for 10 min at 18 °C. An in vitro transcribed 55 nt ^32^P-capped RNA substrate (sequence 5′-G GGCGA ATTGG GCCCG ACGTC GCATG CTCCC GGCCG CCATG GCGGC CGCGG GAAT-3′) and 100 nM SAM were added and the mixture was maintained at 30 °C for 15 min. RNA was purified using phenol–chloroform and precipitated with sodium acetate. RNA was digested with P1 nuclease and cap structures were resolved on PEI cellulose in 0.4 M ammonium sulfate. The migration of caps was visualized by phosphor imaging. Standards were used to verify cap migration.

## Results

### Assay Development

**Figure 1.**
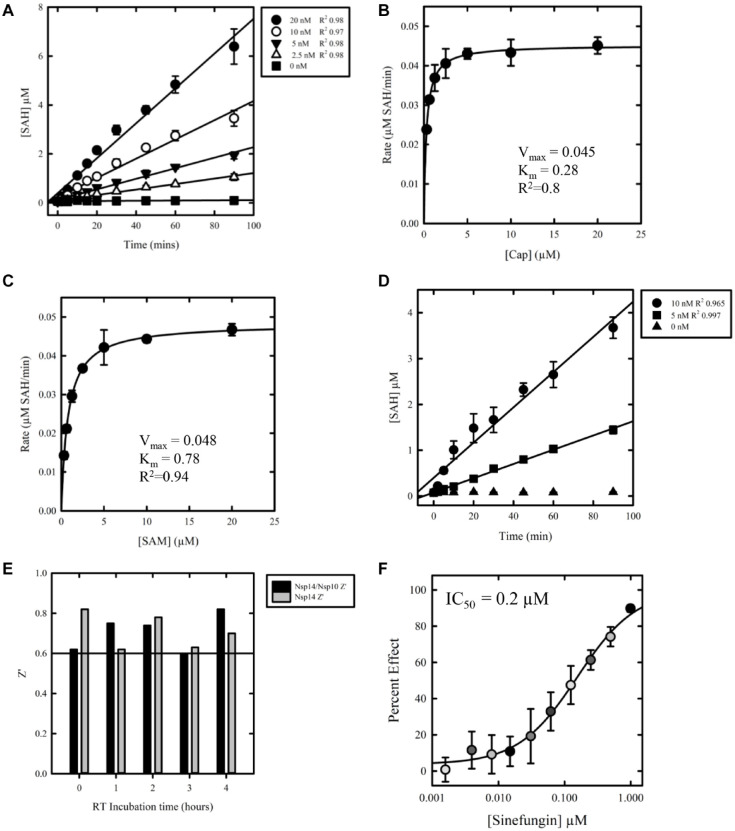
Enzyme kinetics and assay development. Standard curves were used to determine the concentration of SAH produced by reaction. (**A**) Reaction time course up to 90 min to determine initial rates. Nsp14 was tested at 20, 10, 5, and 2.5 nM; 20 µM was the concentration for both SAM and cap. (**B**) Determination of apparent *K*_M_ (*K*_M_^app^) for cap with nsp14. Nsp14 was tested at 10 nM; the cap was titrated from 20 to 0 µM for 60 min. The results are representative of three independent experiments. (**C**) Determination of apparent *K*_M_ (*K*_M_^app^) for SAM with nsp14. Nsp14 was tested at 10 nM; SAM was titrated from 20 to 0 µM for 60 min. The results are representative of three independent experiments. (**D**) Determination of the incubation time range and nsp14 concentration using *K*_M_^app^ for both cap and SAM. Nsp14 was tested at 10 and 5 nM. (**E**) Comparison of reagent stability following preincubation at a range of time points prior to the assay, with or without the addition of nsp10. The impact on assay stability was determined by comparison of Z′. (**F**) Dose–response testing of sinefungin against nsp14 using established screening conditions. Compound was tested at 10 concentrations, 1:2 from 100 µM (top concentration). Data are shown as mean ± SD. The IC_50_ value was 0.2 µM (0.1–0.3 µM; *n* = 4).

The nsp14 enzyme concentration range that linearly correlated with the initial velocity was determined using 20 µM (saturating) cap and SAM. A linear relationship with initial velocity was established up to 30 min for all enzyme concentrations screened (2.5–20 nM) (**Fig. 1A and Suppl. Fig. S3**). A concentration of 10 nM for nsp14 was selected for characterization of cap and SAM kinetics due to this concentration being relatively low but giving a signal window that is sufficient for observing enzymatic activity. Assuming all of the protein is active, the *k*_cat_ is calculated to be 0.088 per second. The *K*_M_^app^ values of the substrates cap and SAM were determined as 0.23 µM (0.15–0.35 µM; *n* = 3) and 0.69 µM (0.51–0.92 µM; *n* = 3), respectively (**Fig. 1B,C**). Typically, substrate concentrations would be screened at close to *K*_M_, as this provides assay conditions suitable for identifying the widest range of inhibitor modalities. Hence, concentrations of 0.7 µM cap and 1.0 µM SAM were selected for use in the assay as being close to *K*_M_ and giving a robust signal (signal-to-background ratio > 5 and Z′ > 0.6) suitable for screening (where Z′ = 1 – ((3 × σ_high signal_ + 3 × σ_low signal_)/(µ_high signal_ – µ_low signal_))).

In order to establish screening conditions for nsp14 under the *K*_M_ conditions above, 5 and 10 nM nsp14 were tested at a range of time points, with 5 nM nsp14 being linear up to 90 min (**Fig. 1D**). Therefore, a 60 min incubation time and 5 nM nsp14 were selected for use in our screen as we were within the linear range of the assay and had a signal-to-background greater than 5, with a Z′ greater than 0.6.

As our screening compounds are solubilized in DMSO, the tolerance of the enzyme assay to this solvent was assessed. Industry standard assays require a tolerance of at least 0.5% DMSO.^22^ Using the established enzymatic conditions, DMSO tolerance was evaluated up to 5% DMSO. Our assay was found to be tolerant up to approximately 2% DMSO (**Suppl. Fig. S4**). At 5% DMSO, the nsp14 retained 70% activity relative to 0% DMSO control.

Nsp14 MTase activity is reported to be independent of nsp10.^23^ In order to confirm this, we performed a titration of nsp10 (0–20 nM) using the established assay conditions described above. We found that neither increasing concentrations of nsp10 (**Suppl. Fig. S5**) nor extended preincubation of nsp10 with nsp14 (**Fig. 1E**) had any effect on the assay.

### Assay Robustness

To assess assay robustness and the signal-to-noise ratio, a mock screen without inhibitors was performed. Two 384-well plates were used, and the last column in both cases lacked the enzyme. The robustness Z′ values were 0.66 and 0.63, and the signal-to-background ratios were 9.1 and 8.4, respectively. In addition, the overall plate coefficients of variation for wells containing nsp14 and substrates were 7.8% and 8.0%. These values are acceptable for high-throughput screening.

Additionally, to evaluate what type of undesirable screening compounds our assay was susceptible to picking up as false positives, we screened our interference set, which consists of 24 compounds with known liabilities: redox cyclers, aggregators, metal chelators and other compounds that are known to interfere with a variety of assay technologies (see **Suppl. Fig. S6A**). The set was screened at 10 µM under the assay conditions identified above. Only one compound, *N*-(3-fluorophenyl)maleimide, resulted in a percentage effect greater than 50% (**Suppl. Fig. S6B**); various other covalent inhibitors did not produce a statistically significant effect. As the compound screening collections that we planned to use in our high-throughput screen are devoid of covalent inhibitors, we were not concerned with this result.

### Assay Validation

In order to validate the nsp14 MS-based assay we have developed, we performed a dose–response study with a known MTase inhibitor, sinefungin (**Fig. 1F**). Sinefungin inhibited nsp14 with an IC_50_ of 0.2 µM (0.1–0.3 µM; *n* = 4).

### Primary Screen

**Figure 2.**
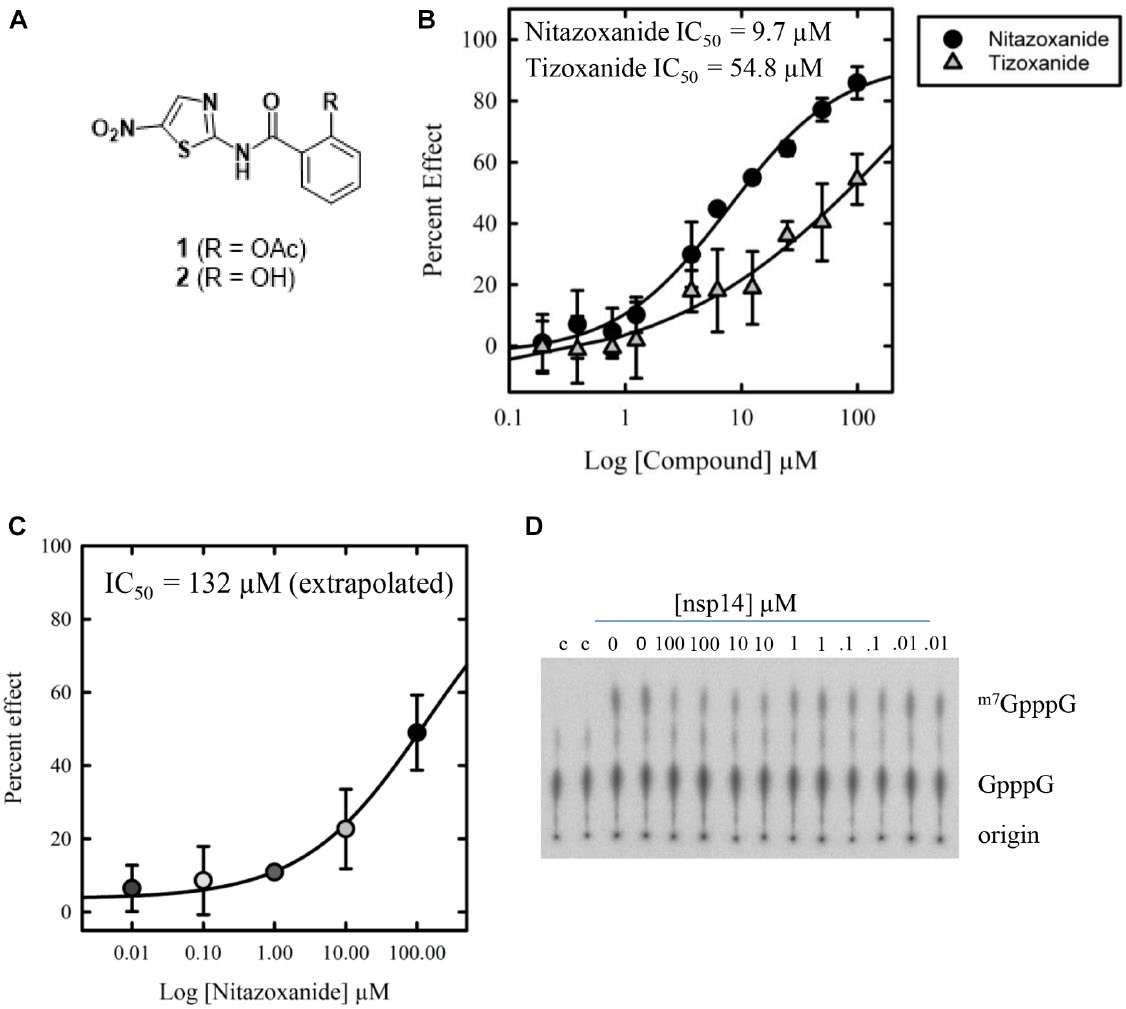
Chemical structure and dose response of nitazoxanide against nsp14. (**A**) Chemical structures of nitazoxanide (**1**) and tizoxanide (**2**). (**B**) Dose–response testing of nitazoxanide and tizoxanide against nsp14 in a primary screening assay. Compounds were tested at 10 concentrations, 1:2 from 100 µM (top concentration). The IC_50_ of nitazoxanide was 9.7 µM (6.3-15.1 µM; *n* = 4), and the IC_50_ of tizoxanide was 54.8 µM (27.3–110.0 µM; *n* = 4). (**C**) Dose–response testing of nitazoxanide against nsp14 in an orthogonal radiometric assay with a representative phosphoimage. Data are shown as mean ± SD.

Having optimized assay conditions, we proceeded to screen the 1771 compounds in the DiscoveryProbe FDA-approved drug library against SARS-CoV-2 nsp14 at a single concentration of 10 µM. The assay performed robustly, with an average Z′ of 0.78 (± 0.02) and an average signal-to-background ratio of 10.45 (the signal-to-background ratio varied between 9.7 and 10.94). The mean percent effect detected was 0.57 with an SD of 8.57. The screen identified 12 hits (hit rate of 0.7%), with hits defined as compounds producing >26% effect on nsp14 activity (mean + 3 SD). All but one of these putative hits were progressed to a full dose response, with nine showing a half maximal inhibitory concentration (IC_50_) of <100 µM (**Suppl. Table 1**). Included in this set were the natural flavonols kaempferol and 3′-hydroxykaempferol, which produced IC_50_ values of 28 µM (25.1–31.6 µM; *n* = 2) and 20 µM (15.8–25.1 µM; *n* = 2), respectively. These compounds have previously been shown to inhibit SARS-CoV-1 nsp14.^21^ The most potent compound was nitazoxanide (**1**) (**Fig. 2A**), which had an IC_50_ against SARS-CoV-2 nsp14 of 9.7 µM (6.3–15.1 µM; *n* = 4) (**Fig. 2B**) and a maximal effect of 89.7%. This compound is of interest as it is already being explored as a potential therapy for COVID-19. In order to determine whether this compound specifically targets the viral RNA cap MTase of SARS-CoV-2, we screened it against the human homolog RNA RNMT, under similar conditions to those described above. Nitazoxanide showed no activity within the concentration range screened (IC_50_ > 100 µM).

Following oral administration, nitazoxanide is rapidly hydrolyzed to the pharmacologically active metabolite, tizoxanide (**2**) (**Fig. 2A**),^24^ which has not yet been studied for anti-SARS-CoV-2 activity. We therefore profiled tizoxanide under the assay conditions described above. Tizoxanide produced an IC_50_ against SARS-CoV-2 nsp14 of 54.8 µM (27.3–110.0 µM; *n* = 4) and a maximal effect of 62.0%.

### Orthogonal Screen

In order to validate the nitazoxanide inhibition of nsp14 observed in our high-throughput MS assay, we screened nitazoxanide in an MTase activity assay that utilizes guanosine-capped RNA as a substrate. In this assay, a titration of nitazoxanide was incubated with nsp14 prior to the addition of the ^32^P-guanosine-capped substrate and SAM. Following the methylation reaction, RNA was purified and digested by P1 nuclease to release cap structures, which were resolved by thin-layer chromatography. Forty-nine percent inhibition of N7-guanosine cap methylation was observed at 100 µM (**Fig. 2C**). Based on a titration of nitazoxanide (0.01–100 µM) under the assay conditions described above, we project an IC_50_ of 132 µM.

## Discussion

The identification of inhibitors of SARS-CoV-2 is of significant global interest at present. We, like others, believe that the quickest way to identify small-molecule inhibitors of this deadly virus, and address the current global health emergency, is to repurpose approved drugs developed for other uses. This approach allows the global community to take advantage of existing information on human pharmacology and toxicology, enabling rapid clinical trials.

Herein, we have reported the development of a screening platform to identify small-molecule inhibitors of the N7-MTase activity of nsp14, a key enzyme involved in generating RNA cap structures and essential in masking viral RNA from the host immune system. Our high-throughput MS-based assay allows direct, rapid, and label-free measurement of SAH, the by-product from the nsp14-mediated transfer of a methyl group from *S*-adenosyl-l-methionine (SAM). Compared with traditional fluorescence assays, MS-based assays are more sensitive and require less enzyme (reducing cost and the tight binding limit), have higher a signal-to-noise ratio, and produce more robust Z′. In addition, the direct detection of reaction product significantly minimizes the risk of identifying false-positive hits. Using this technology, we screened ApexBio’s DiscoveryProbe screening library, a chemically diverse set of 1771 FDA-approved small-molecule drugs, at a single concentration of 10 µM. Under our optimized conditions, our assay performed robustly, with an average Z′ of 0.78 (± 0.02) and an average signal-to-background of 10.45. The hit rate was 0.7%, and we identified 12 statistically significant hits, the most interesting of which was nitazoxanide. Nitazoxanide produced an IC_50_ against SARS-CoV-2 nsp14 of 9.7 µM with a maximal effect of 92.1% and was selective for viral enzyme over the human homolog, RNMT (IC_50_ > 100 µM). Activity was confirmed in a cap MTase activity assay that directly measures methyltransfer to a 55 nt transcript. The 10-fold shift in activity observed between this assay and our primary biochemical assay could be the result of different assay conditions. It has, however, been observed that compounds that bind to the RNA-binding site of the enzyme show diminished activity when longer RNA substrates are used.^25^ Mode of inhibition studies would be useful to confirm whether nitazoxanide is indeed a cap competitive inhibitor.

Nitazoxanide is a commercial anti-infective agent with efficacy in parasitic, bacterial, and viral infections. Nitazoxanide has antiviral activity against a range of human and animal coronaviruses, including Middle East respiratory syndrome coronavirus (MERS-CoV),^26^ and has recently been shown to be able to block SARS-CoV-2 in vitro infections at low micromolar concentrations (EC_50_ = 2.12 µM).^27^ With MERS-CoV, nitazoxanide acts by blocking maturation of the viral nucleocapsid N protein that promotes production of the viral particles. Nitazoxanide is relatively safe in humans, and studies showed tolerability of single doses up to 4 g with minimal gastrointestinal side effects. This drug is currently being tested in a clinical trial (NCT04341493) as a stand-alone therapy (500 mg BID) and in combination with hydroxychloroquine for the treatment of COVID-19.

It is unlikely that the inhibition of viral replication seen in the cell-based assays described above is solely driven by the N7-MTase activity of nitazoxanide. These results have, however, identified a validated and selective chemical inhibitor of this important viral target, and this compound could serve as a starting point for further optimization.

## Supplemental Material

sj-pdf-1-jbx-10.1177_24725552211000652 – Supplemental material for Development of a High-Throughput Screening Assay to Identify Inhibitors of the SARS-CoV-2 Guanine-N7-Methyltransferase Using RapidFire Mass Spectrometry Click here for additional data file. Supplemental material, sj-pdf-1-jbx-10.1177_24725552211000652 for Development of a High-Throughput Screening Assay to Identify Inhibitors of the SARS-CoV-2 Guanine-N7-Methyltransferase Using RapidFire Mass Spectrometry by Lesley-Anne Pearson, Charlotte J. Green, De Lin, Alain-Pierre Petit, David W. Gray, Victoria H. Cowling and Euan A. F. Fordyce in SLAS Discovery
