# Supplementary material for: Development of a High-Throughput Screening Assay to Identify Inhibitors of the SARS-CoV-2 Guanine-N7-Methyltransferase Using RapidFire Mass Spectrometry
Source: SLAS Discov. 2021 Mar 16;26(6):749–56. doi: 10.1177/24725552211000652 (PMC7967019; doi:10.1177/24725552211000652)
Supplement: sj-pdf-1-jbx-10.1177_24725552211000652 – Supplemental material for Development of a High-Throughput Screening Assay to Identify Inhibitors of the SARS-CoV-2 Guanine-N7-Methyltransferase Using RapidFire Mass Spectrometry [file sj-pdf-1-jbx-10.1177_24725552211000652.pdf]

Supplementary Material

Development of a high-throughput screening assay to identify inhibitors of the SARS-CoV-2 guanine-N7 methyltransferase using RapidFire mass spectrometry

Lesley-Anne Pearson,[a] Charlotte J. Green,[a] De Lin,[a] Alain-Pierre Petit,[a] David W. Gray,[a*] Victoria H. Cowling,[b] Euan A. F. Fordyce[a*]

a. Drug Discovery Unit, School of Life Sciences, University of Dundee, Dundee, DD1 5EH, United Kingdom
b. Centre for Gene Regulation and Expression, School of Life Sciences, University of Dundee, Dundee, DD1 5EH, United Kingdom

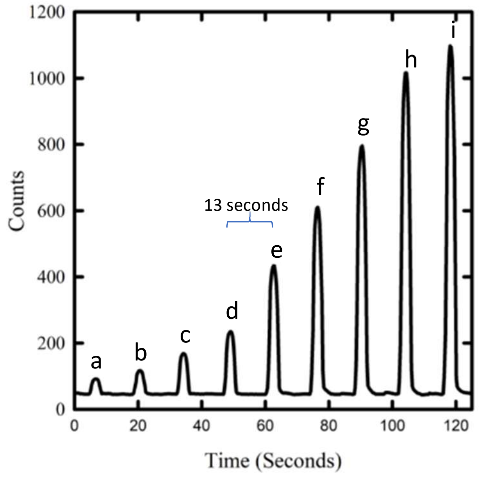

Supplemental Figure 1:
Representative chromatogram for RF-MS/MS (MRM transition 385.1/134) of SAH formation in the reaction of 5 nM nsp14, 1 μM SAM and 0.7 μM cap at various time points. (a) 0 min; (b) 2 min; (c) 5 min; (d) 10 min; (e) 20 min; (f) 30 min; (g) 45 min; (h) 60 min; (i) 90 min. The injection cycle time is 13 seconds.

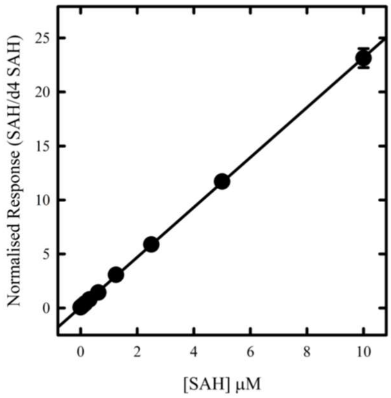

Supplemental Figure 2:
Representative calibration curve (standard curve) for SAH by RF-MS/MS. SAH was spiked at 0.017 – 10 μM in a background of assay buffer and quenching solution containing 0.08 μM internal standard (d4 SAH) and measured by RF-MS/MS. Values plotted are mean ± standard deviation (n=4)

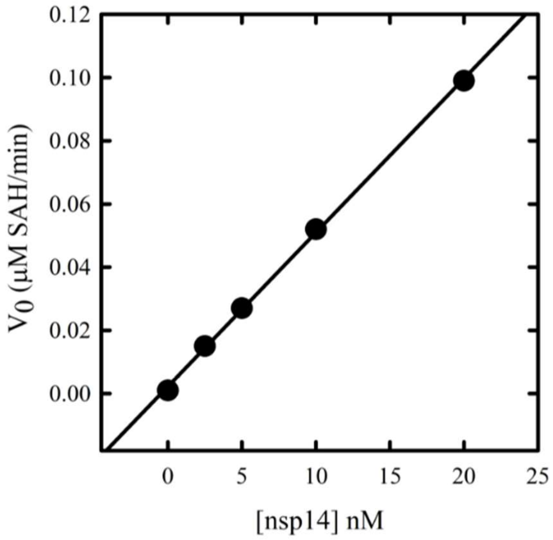

Supplemental Figure 3:
Plot of initial velocity against nsp14 concentration. Initial velocity determined up to 30 minutes.

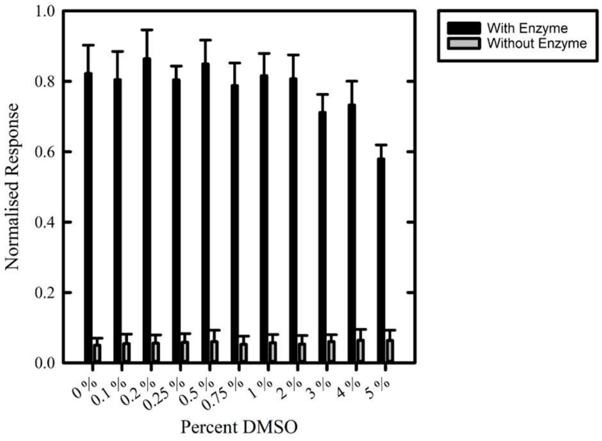

Supplemental Figure 4:
Determination of nsp14 sensitivity to DMSO. Nsp14, cap and SAM tested at identified screening concentrations (5 nM, 0.7 μM and 1 μM respectively) for 60 minutes.

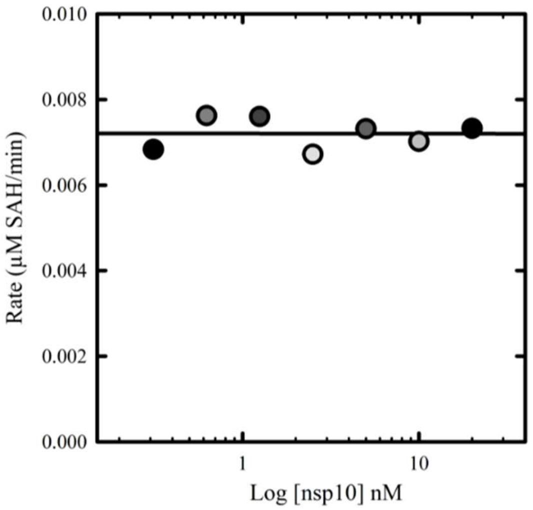

Supplemental Figure 5
Determination of effect of increasing nsp10 concentrations on reaction rate. Nsp10 titrated from a top concentration of 20 nM with identified screening concentrations of all other assay components. Rates determined from reaction progress curves.

A

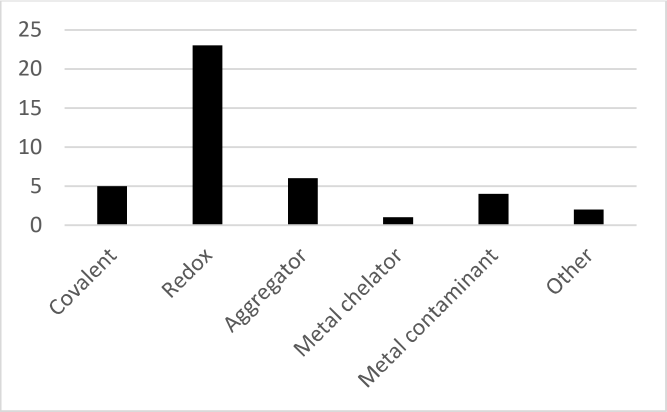

B

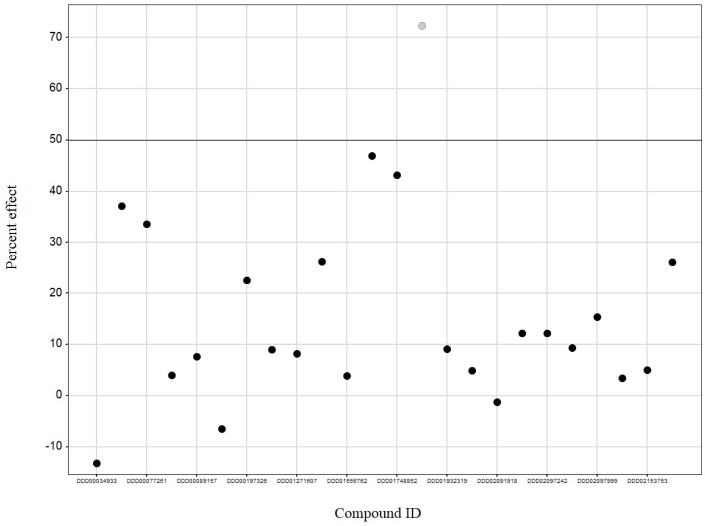

Supplemental Figure 6:
A) Breakdown on the number of compounds for each assay interference mechanism and B) Plot of results from interference set testing. Cut off was set at 50 % (grey line).

Name	Structure	SARS-CoV-2 nsp14 IC_{50} (n = 2)
Balsalazide	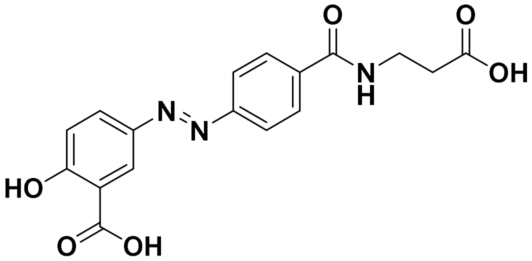	22.8 - >100 μM
Bendamustine	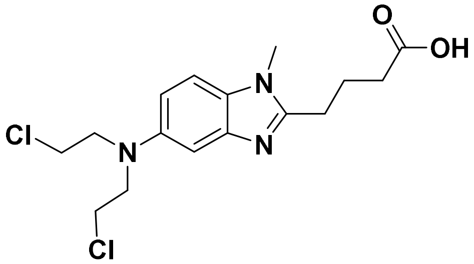	26.6 - 40.1 μM
Diacerein	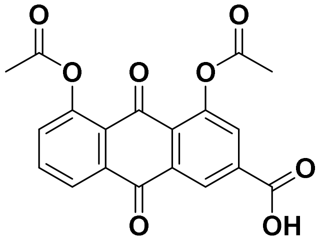	40.7 - 80.4 μM
3’-hydroxylkaempferol	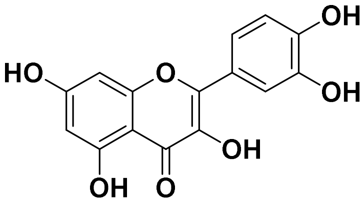	16.9 - 22.8 μM
Kaempferol	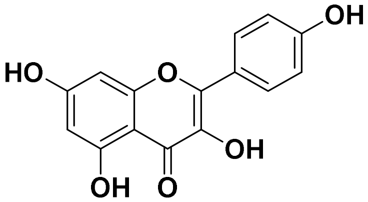	22.4 - 28.5 μM
Omipalisib	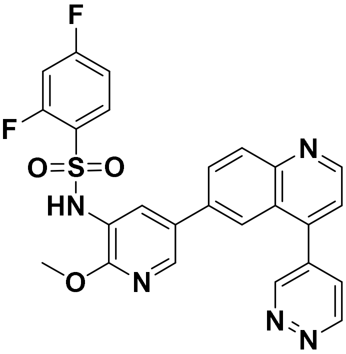	5.5 - 55.0 μM

Phenoxybenzamine	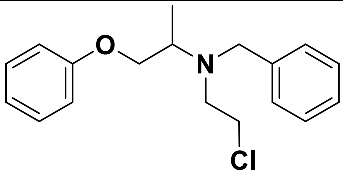	58.2 - 91.0 µM
Prasugrel	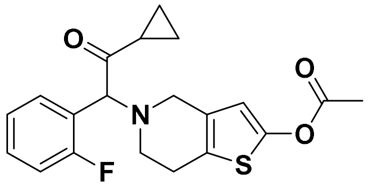	25.8 - 36.6 µM

Supplemental Table 1:
Names, structures and nsp14 IC_{50} for statistically significant hits
